# Early Reduction in Mitochondrial Membrane Potential in Synaptic Mitochondria Contribute to Synaptic Pathology in the EAE Mouse Model of Multiple Sclerosis

**DOI:** 10.3390/ijms27062579

**Published:** 2026-03-11

**Authors:** Dalia R. Ibrahim, Karin Schwarz, Ajay Kesharwani, René Tinschert, Shweta Suiwal, Frank Schmitz

**Affiliations:** Department of Neuroanatomy, Institute of Anatomy and Cell Biology, Saarland University, 66421 Homburg, Germany; daliaibrahim93@outlook.com (D.R.I.); karin.schwarz@uks.eu (K.S.); biotechajay@gmail.com (A.K.); tinschert.rene@gmail.com (R.T.); shwetasuiwal@gmail.com (S.S.)

**Keywords:** multiple sclerosis, ribbon synapse, retina, mitochondria, MIC60, mitochondrial membrane potential, EAE

## Abstract

Multiple sclerosis (MS) is a highly disabling chronic autoimmune disease of the central nervous system with neuroinflammatory and neurodegenerative alterations found in the white and grey matter of the brain. The pathogenesis of MS is complex and not fully understood. Mitochondrial dysfunctions are suspected to play an important role. The visual system is often affected in MS. Optic neuritis is a frequent symptom, but also the retina itself, including retinal synapses appear compromised in MS independent from demyelination of the optic nerve. A previous study demonstrated synapse-specific alterations of mitochondria in photoreceptor synapses in the Experimental Autoimmune Encephalomyelitis (EAE) mouse model of MS at day 9 after injection, an early time point in pre-clinical EAE. In the present study, we analysed even earlier stages of pre-clinical EAE for possible alterations of synaptic mitochondria. For this purpose, we performed qualitative and quantitative immunolabelling analyses of the mitochondrial cristae organising protein MIC60 at retinal synapses and functional analyses by measuring synaptic mitochondrial membrane potential (during rest and depolarisation-induced exocytosis) and visually guided behaviour (optometry analyses). At day 3 after injection, morphological and functional data were indistinguishable between MOG/CFA-injected EAE mice and CFA-injected control mice. But already on day 5 after injection, we observed a decreased expression of the mitochondrial MIC60 protein at synaptic mitochondria, a decreased synaptic mitochondrial membrane potential at rest, an enhanced drop of mitochondrial membrane potential during stimulated exocytosis and a decreased visual performance of the respective EAE mice. These data argue that synaptic pathology in the EAE retina begins as early as day 5 after injection. Our data propose that dysfunctions of mitochondria play an important role already at the very early stages of synaptic pathology in EAE.

## 1. Introduction

Multiple sclerosis (MS) is a chronic inflammatory autoimmune disease of the central nervous system (CNS) that results in multifaceted pathological alterations in the white and grey matter of the brain, with rising incidence in the world population [1,2,3,4,5,6,7,8,9,10,11]. In more advanced stages of the disease, myelinated axons in the white matter are destroyed and replaced by glial scars. Independent from demyelination, early alterations also occur in the grey matter of the CNS and include neurodegeneration [6] and changes in synapse structure and function [7,8,9,11]. Synaptic dysfunctions already occur at early stages of the disease, as also observed in animal models of multiple sclerosis, e.g., the Experimental Autoimmune Encephalomyelitis (EAE) model [6,8,9,10]. In the EAE animal model, EAE is basically induced by subcutaneous injection of the encephalitogenic myelin oligodendrocyte glycoprotein (MOG) peptide, dissolved in complete Freund’s adjuvant (CFA), followed by intraperitoneal pertussis toxin injections mainly to weaken the blood–brain barrier [12,13,14,15,16,17,18,19,20,21,22,23,24]. CFA-only-injected mice (i.e., without MOG peptide) but with pertussis toxin injections serve as controls.

The CNS, which includes the retina, has a very high energy demand [25,26,27,28,29,30,31,32]. Most energy consumed by the CNS is used for synaptic transmission, and mitochondria are considered vital energy-producing organelles to maintain synaptic transmission [26,30,33,34,35,36,37,38,39,40,41,42,43,44,45,46,47]. In neurons, about 90% of total ATP is produced by mitochondria [26,30,31]. Mitochondrial dysfunctions and disorders of synapse energetics are also suspected to play an important role in the pathogenesis of MS [48,49,50,51,52,53,54,55,56,57,58,59,60,61,62,63,64,65].

The visual system is frequently impaired in MS [24,66,67,68,69,70]. Optic neuritis is an early symptom in many MS patients [71,72,73]. The retina itself is also altered in MS and animal models of MS [74,75,76,77,78,79,80,81,82,83,84]. In the retina, photoreceptors and retinal bipolar cells form glutamatergic ribbon synapses [85,86,87,88,89,90,91,92], and retinal ribbon synapses are affected in the EAE mouse model of MS [75,76,77,78,79,81,82,84]. In a previous study, we showed alterations of functionally relevant mitochondrial proteins in mitochondria of photoreceptor synapses from EAE mice on day 9 after injection [84]. These alterations were specific to synaptic mitochondria. We demonstrated that mitochondria located at different subcellular locations within the same cell, i.e., the photoreceptor cell, were functionally heterogeneous [84]. Mitochondria in the presynaptic terminals of photoreceptor synapses, which are located close to the synaptic ribbon, were strongly impaired in EAE at day 9 after injection. These synaptic mitochondria showed a decreased expression of the mitochondrial cristae organising protein MIC60 at that early time point in the pre-clinical phase of EAE [84]. In contrast, mitochondria in the inner segments of the same cell were not affected by EAE [84]. The mouse retina is a rod-dominated retina with more than 95% of the photoreceptors being rod photoreceptors [92,93]. Remarkably, rod photoreceptor presynaptic terminals in the mouse retina contain a single, very large presynaptic mitochondrion in close vicinity to the synaptic ribbon that is anchored to the active zone neurotransmitter release sites [84,94,95,96,97,98]. This large presynaptic mitochondrion appears to be ideally placed for local ATP/energy production to rapidly support energy-demanding synaptic transmission at the continuously active rod photoreceptor ribbon synapse. In the present study, we tested whether mitochondrial alterations in the presynaptic photoreceptor terminals even occurred at an earlier time point before day 9 after injection, i.e., day 3 and day 5 after injection, and, if yes, whether these are associated with functional alterations of the photoreceptor synaptic mitochondria.

## 2. Results

In a previous study [84], we identified alterations in the protein composition of synaptic mitochondria of retinal photoreceptor synapses at day 9 after injection in EAE mice. We demonstrated that the mitochondrial protein MIC60, an essential organiser of the inner mitochondrial membrane [99,100], is specifically altered in synaptic mitochondria of photoreceptors in MOG/CFA-injected EAE mice on day 9 after injection, whereas mitochondria within the same cell but at other non-synaptic intracellular locations were unaffected [84]. In the current study, we tested whether MIC60 could be altered in synaptic mitochondria in the OPL even at an earlier time point than day 9 after injection.

**Figure 1:**
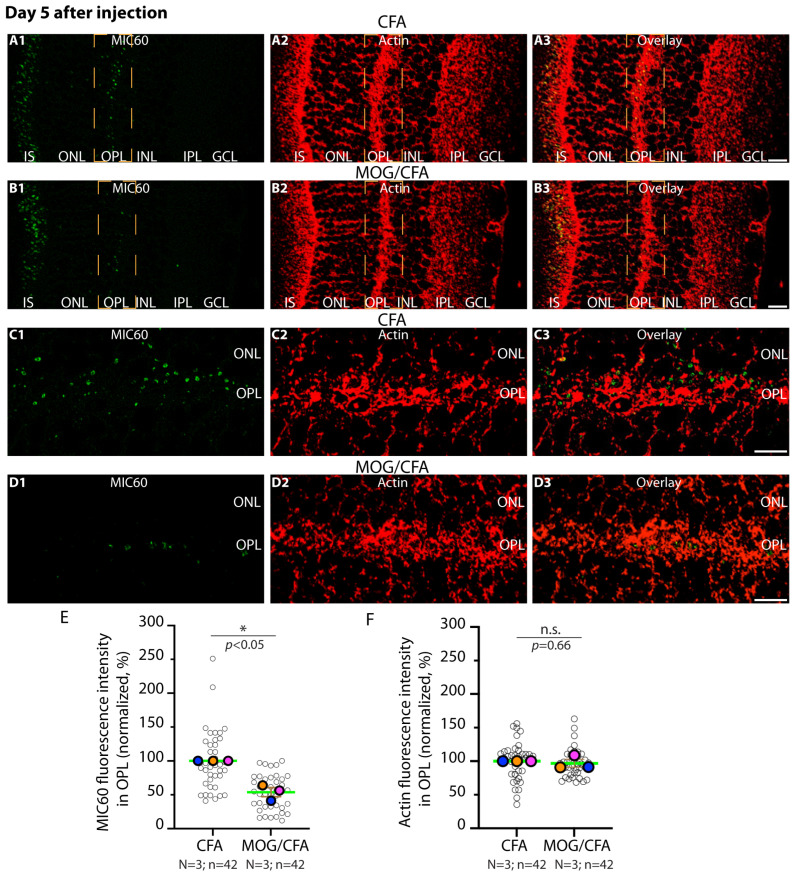
**Decreased expression of MIC60 in synaptic mitochondria of photoreceptor ribbon synapses in the OPL of the retina in MOG/CFA-injected EAE mice in comparison to CFA-injected control mice at day 5 after injection.** 0.5 µm thin retinal sections from CFA control mice (**A1**–**A3**,**C1**–**C3**) and EAE mice (**B1**–**B3**,**D1**–**D3**) double-immunolabelled with rabbit polyclonal anti-MIC60 antibody and mouse monoclonal anti-actin antibody. Merged images from green (**A1**,**B1**,**C1**,**D1**) and red channels (**A2**,**B2**,**C2**,**D2**) were overlayed in (**A3**,**B3**,**C3**,**D3**). (**A1**–**A3**,**B1**–**B3**) show lower magnified representative images. Higher magnification images from the immunolabelled OPL are shown in (**C1**–**C3**,**D1**–**D3**). An exemplary region-of-interest (ROI) covering the OPL, in which the photoreceptor synapses are located, is schematically plotted in orange with a dashed line in (**A1**–**A3**,**B1**–**B3**). (**E**,**F**) Quantification of MIC60 and actin immunosignals in the OPL (measured as integrated density; data depicted as SuperPlots). MOG/CFA values were normalised to the corresponding actin signal in the OPL and related to the arithmetic mean of CFA, which was set to 100% in each independent experiment (**E**,**F**). The Superplots show the arithmetic means (green horizontal lines) ± S.E.M.s. Filled colored circles represent the arithmetic means of the individual independent experiments; the open circles show the individual datapoints from all experiments. One-sample *t*-tests were used for statistical analyses in (**E**,**F**). *p*-values < 0.05 were considered statistically significant. Abbreviations: CFA, complete Freund’s adjuvant; MOG, myelin oligodendrocyte glycoprotein; EAE, experimental autoimmune encephalomyelitis; IS, inner segment; OPL, outer plexiform layer; ONL, outer nuclear layer; INL, inner nuclear layer; IPL, inner plexiform layer; GCL, ganglion cell layer; S.E.M., standard error of the mean; N = number of independent experiments; n = number of analysed images; n.s., non-significant; *, *p* < 0.05. Scale bars: 5 μm.

First, we tested whether MIC60 could already be altered at day 5 after injection in the early, pre-clinical phase of EAE. For this purpose, we performed double immunolabelling experiments with antigen-affinity-purified rabbit antibody against MIC60 and mouse monoclonal antibody against actin (clone C4) on the respective retina samples (Figure 1). Actin served as a reference protein, as in our previous studies [75,78,81,84].

**Figure 2:**
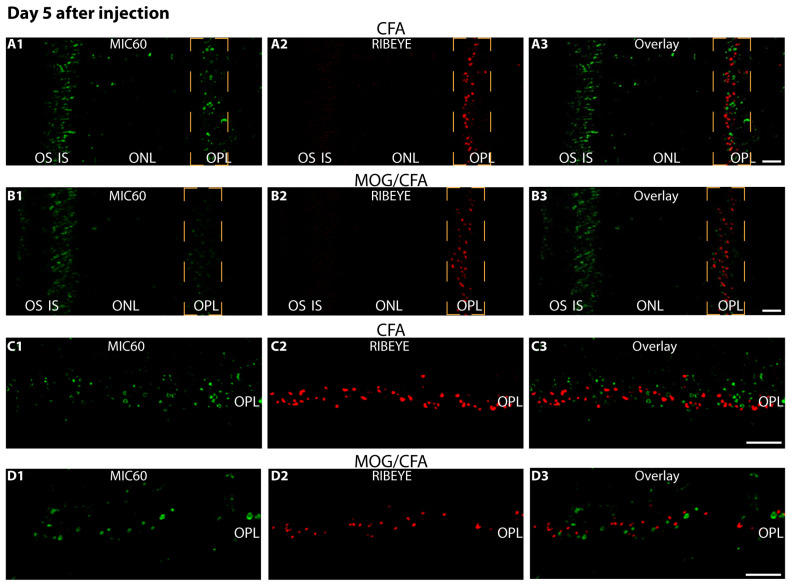

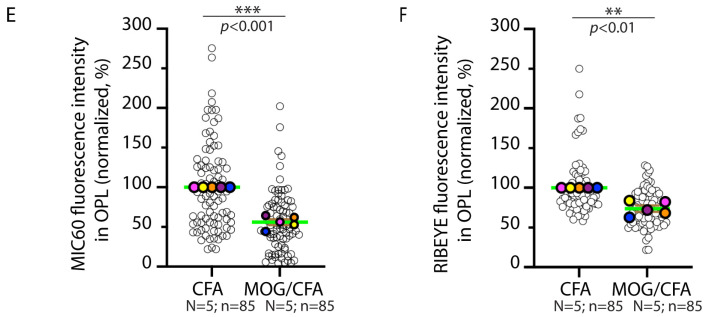
**Decreased expression of MIC60 and RIBEYE in photoreceptor ribbon synapses in the OPL of the retina in MOG/CFA-injected EAE mice in comparison to CFA-injected control mice at day 5 after injection.** The 0.5 µm thin retinal sections from CFA control mice (**A1**–**A3**,**C1**–**C3**) and EAE mice (**B1**–**B3**,**D1**–**D3**) were double-immunolabelled with rabbit polyclonal anti-MIC60 antibody and mouse monoclonal anti-RIBEYE antibody (clone 2D9). Merged images from green (**A1**,**B1**,**C1**,**D1**) and red channels (**A2**,**B2**,**C2**,**D2**) were overlayed in (**A3**,**B3**,**C3**,**D3**). (**A1**–**A3**,**B1**–**B3**) shows lower magnified representative images. Higher magnification images from the immunolabelled OPL are shown in (**C1–C3,D1–D3**). An exemplary region-of-interest (ROI) covering the OPL, in which the photoreceptor synapses are located, is schematically plotted in orange with a dashed line in (**A1**–**A3**,**B1**–**B3**). (**E**,**F**) Quantification of MIC60 and RIBEYE immunosignals in the OPL (measured as integrated density; data depicted as SuperPlots). MOG/CFA values were normalised to the arithmetic means of CFA, which were set to 100% in each independent experiment (**E**,**F**). The Superplots show the arithmetic means (green horizontal lines) ± S.E.M.s. Filled circles represent the arithmetic means of the individual independent experiments; the open circles show the individual datapoints from all experiments. One-sample *t*-tests were used for statistical analyses in (**E**,**F**). Abbreviations: CFA, complete Freund’s adjuvant; MOG, myelin oligodendrocyte glycoprotein; EAE, experimental autoimmune encephalomyelitis; OS, outer segment; IS, inner segment; OPL, outer plexiform layer; ONL, outer nuclear layer; S.E.M., standard error of the mean; N = number of independent experiments; n = number of analysed images; ***, *p* < 0.001 **, *p* < 0.01. Scale bars: 5 μm.

On day 5 after injection, we observed a decreased expression of MIC60 in synaptic mitochondria of photoreceptor synapses in the OPL (Figure 1(A1–A3,B1–B3,C1–C3,D1–D3,E)). Actin immunosignals were unchanged in MOG/CFA-injected mice in comparison to control littermate mice (CFA-injected mice) on day 5 after immunisation (Figure 1(A1–A3,B1–B3,C1–C3,D1–D3,F)), indicating that there is no global decrease in protein expression and no global cell death/tissue atrophy, similarly to what was observed in previous studies at even later time points of pre-clinical EAE [75,78,81,84]. Similar results were obtained by double-immunolabelling retinal sections of MOG/CFA EAE mice and CFA control mice with antigen-affinity-purified rabbit antibody against MIC60 and mouse monoclonal antibody against RIBEYE (clone 2D9), the main component of synaptic ribbons (Figure 2). Again, we observed a decrease in the strength of the immunosignals for MIC60 in the OPL, where the synaptic mitochondria of photoreceptor terminals are located (Figure 2(A1–A3,B1–B3,C1–C3,D1–D3,E)). Interestingly, we also observed a decrease in the strength of the RIBEYE immunosignal in the OPL at day 5 after injection (Figure 2(A1–A3,B1–B3,C1–C3,D1–D3,F)). A decrease in RIBEYE immunosignals in the OPL was previously observed on day 9 after injection [75,78]. This indicates that the synaptic ribbons are already altered not only at day 9 after injection but also at day 5 after injection.

Thus, changes in mitochondrial MIC60 and RIBEYE were already present in the OPL at day 5 after injection in EAE mice and not only at later stages, i.e., day 9 after injection, as previously described [75,84]. Of note, day 9 after injection is still within the pre-clinical phase of EAE in which no scorable symptoms as recorded in the classical score sheets (e.g., limb paralysis) were observed.

**Figure 3:**
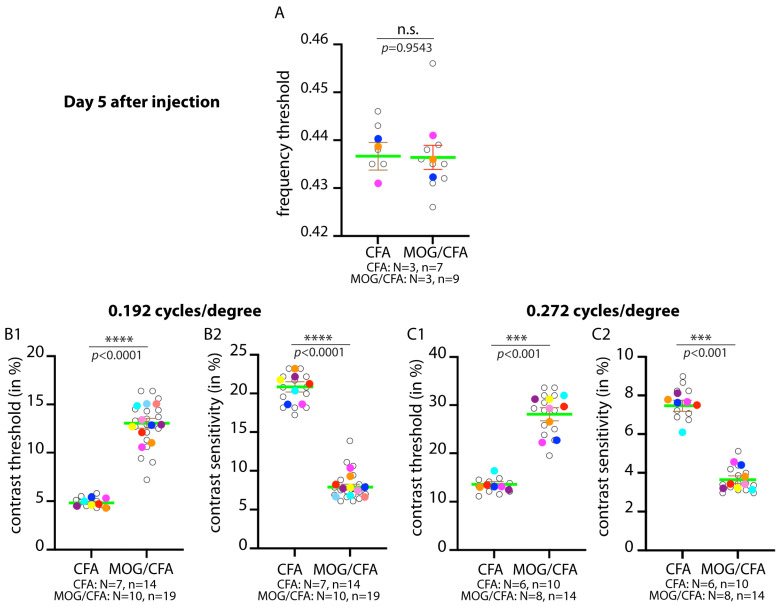
**Differences in optokinetic responses on day 5 after injection in MOG/CFA-injected EAE mice in comparison to CFA-injected control mice.** Optokinetic responses were recorded from MOG/CFA-injected EAE mice on day 5 after injection and analysed for frequency thresholds (**A**) and contrast sensitivity/threshold at two different rotation speeds: 0.192 cycles/degree (**B1**,**B2**) and 0.272 cycles/degree (**C1**,**C2**). Data are depicted as SuperPlots. The SuperPlots show the arithmetic means (green horizontal lines) ± S.E.M.s. Filled circles represent the arithmetic means of the individual independent experiments; the open circles show the individual datapoints from all experiments. Welch’s *t*-test (*t*-test with Welch’s correction) was used for statistical analyses in (**A**,**B1**,**B2**); Mann–Whitney test for (**C1**,**C2**). *p*-values < 0.05 were considered statistically significant. Abbreviations: EAE, experimental autoimmune encephalomyelitis; S.E.M., standard error of the mean; N, number of independent mice; n = number of independent measurements; ****, *p* < 0.0001; ***, *p* < 0.001; n.s., non-significant.

The present data indicate that the synaptic pathology, as judged by decreased expression of MIC60 and RIBEYE, even starts earlier, i.e., at day 5 after injection in EAE mice. Supported by these morphological data, we checked whether functional changes already exist at this early stage of pre-clinical EAE in comparison to littermate controls. For this purpose, we performed measurement of the optokinetic reflex with an optometry setup, as previously described [75,101]. First, we determined the frequency threshold of visual detection in the optometry experiments. We found that the frequency threshold of visual detection was identical (at ~0.44 cycles/degree) between MOG/CFA-injected mice and CFA-injected control mice at day 5 after injection (Figure 3A). Previous data showed that the frequency threshold was significantly different between MOG/CFA-injected mice at day 9 after injection [75], indicating that the alterations of visually guided performance are milder at day 5 after injection when compared to day 9 after injection. However, already at day 5 after injection, the contrast sensitivity of MOG/CFA-injected mice at two rotation frequencies (0.192 cycles/degree and 0.272 cycles/degree) was significantly diminished in comparison to the CFA-injected littermate control mice (Figure 3(B2,C2)). Vice versa, the contrast threshold in MOG/CFA-injected EAE mice was higher than in the CFA-injected control mice (Figure 3(B1,C1)).

**Figure 4:**
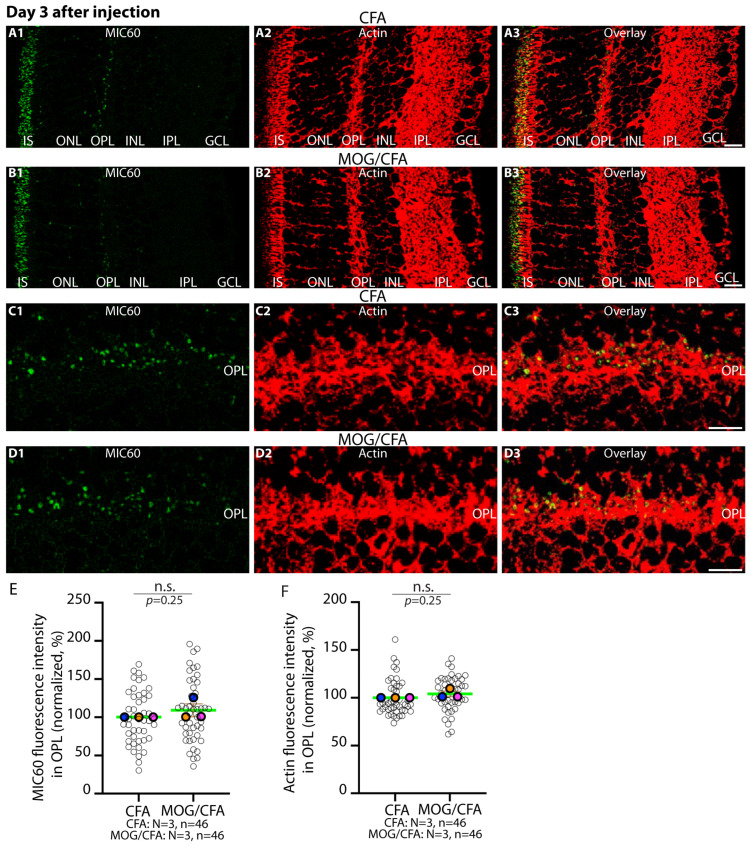
**MIC60 IF immunosignals in the OPL are not altered in MOG/CFA-injected mice in comparison to CFA-injected mice at day 3 after injection.** The 0.5 µm thin retinal sections from CFA control mice (**A1**–**A3**,**C1**–**C3**) and EAE mice (**B1**–**B3**,**D1**–**D3**) obtained at day 3 after injection were double-immunolabelled with rabbit polyclonal anti-MIC60 antibody and mouse monoclonal anti-actin antibody (clone **C4**). Merged images from green (**A1**,**B1**,**C1**,**D1**) and red channels (**A2**,**B2**,**C2**,**D2**) were overlayed in (**A3**,**B3**,**C3**,**D3**). (**A1**–**A3**,**B1**–**B3**) shows representative lower magnified images. Higher magnification images from the immunolabelled OPL are shown in (**C1–C3,D1**–**D3**). (**E**) The MIC60 IF signal strength in the OPL (measured as integrated density) was normalised to the corresponding actin signal in the OPL and related to the arithmetic means of the CFA values, which were set to 100% in each experiment. (**F**) The actin IF signal measured in OPL (as integrated density) served as a reference protein. Data in (**E**,**F**) were depicted as SuperPlots. The SuperPlots show the arithmetic means (green horizontal lines) ± S.E.M.s. Filled circles represent the arithmetic means of the individual independent experiments; the open circles show the individual datapoints from all experiments. Wilcoxon signed-rank tests were used for statistical analysis in (**E**,**F**). *p*-values < 0.05 were considered statistically significant. Abbreviation: CFA, complete Freund’s adjuvant; MOG, myelin oligodendrocyte glycoprotein; EAE, experimental autoimmune encephalomyelitis; IF, immunofluorescence; IS, inner segment; OPL, outer plexiform layer; ONL, outer nuclear layer; INL, inner nuclear layer; IPL, inner plexiform layer; GCL, ganglion cell layer; S.E.M., standard error of the mean; N = number of independent experiments; n = number of analysed images; n.s., non-significant. Scale bars: 5 μm.

Since morphological alterations of photoreceptor synaptic mitochondria and functional alterations in the visual system were already present on day 5 after injection in MOG/CFA-injected EAE mice in comparison to CFA-injected control mice, we tested whether these changes might occur at even earlier time points, i.e., day 3 after injection. First, we tested for the expression of MIC60 in synaptic mitochondria of the OPL in retinas obtained from MOG/CFA-injected EAE animals in comparison to CFA-injected control mice at day 3 after injection (Figure 4). We found that the MIC60 contents of synaptic mitochondria were indistinguishable in the OPL of MOG/CFA-injected mice in comparison to CFA-injected littermate control mice at day 3 after injection (Figure 4).

**Figure 5:**
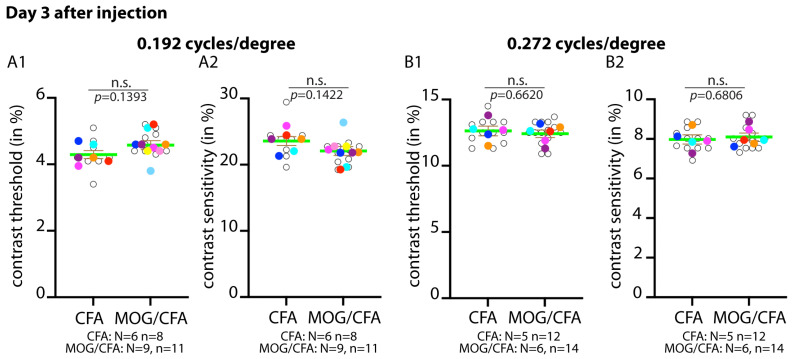
**No differences in optokinetic responses on day 3 after injection in MOG/CFA-injected EAE mice in comparison to CFA-injected control mice on day 3 after injection.** Optokinetic responses were recorded from MOG/CFA-injected mice on day 3 after injection and analysed for contrast sensitivity/threshold at two different rotation speeds: 0.192 cycles/degree (**A1**,**A2**) and 0.272 cycles/degree (**B1**,**B2**). (**A1**,**A2**,**B1**,**B2**) Data are depicted as SuperPlots. The SuperPlots show the arithmetic means (green horizontal lines) ± S.E.M.s. Filled circles represent the arithmetic means of the individual independent experiments; the open circles show the individual datapoints from all experiments. Welch’s *t*-test (*t*-test with Welch’s correction) was used for statistical analyses in (**A1**,**A2**,**B1**,**B2**). *p*-values < 0.05 were considered statistically significant. Abbreviations: S.E.M., standard error of the mean; N, number of independent mice; n = number of independent measurements; n.s., non-significant.

We also performed optometry tests on day 3 after injection (Figure 5). Since the frequency threshold was already unchanged on day 5 after injection, we only performed/analysed contrast threshold/ sensitivity measurements on day 3 after injection at two different frequencies (0.192 cycles/degree; 0.272 cycles/degree) (Figure 5).

Since at day 3 after injection, no differences at the morphological (MIC60 in OPL; Figure 4) and functional levels (optometry; Figure 5) were observed between EAE mice and CFA control mice, we conclude that a synaptic pathology has not yet developed at that time point, i.e., day 3 after injection. Therefore, we focused on day 5 after injection for further functional analyses. Day 5 after injection appears to be the time point at which synaptic pathology starts.

**Figure 6:**
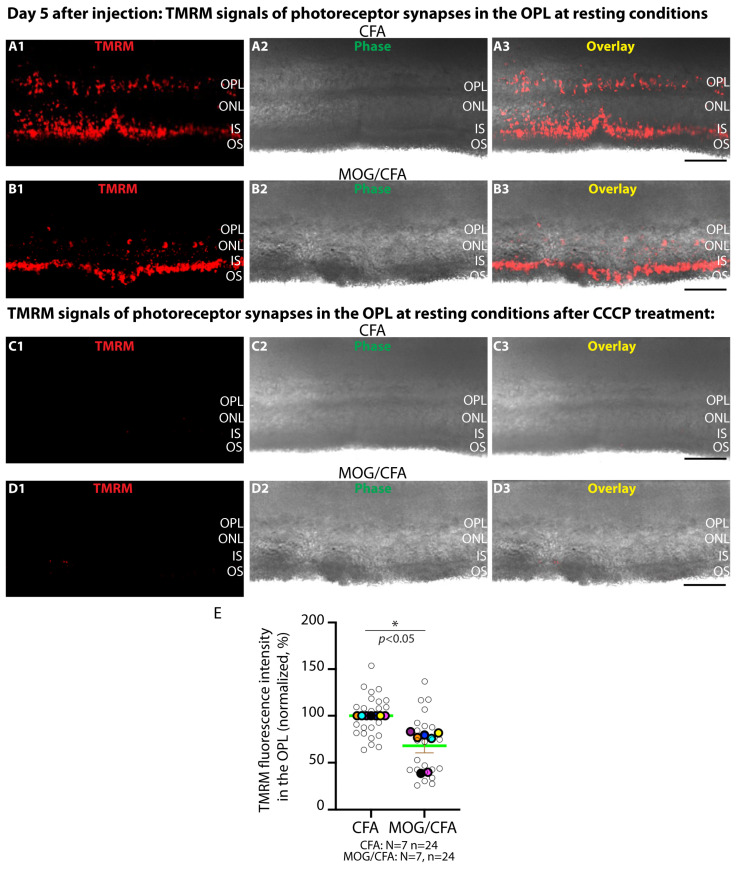
**Decreased TMRM fluorescence signals in the OPL of MOG/CFA-injected EAE mice in comparison to CFA-injected control mice at day 5 after injection under resting conditions.** Representative exemplary images of living retinal slices from CFA control mice (**A1**–**A3**,**C1**–**C3**) and MOG/CFA-injected mice (**B1**–**B3**,**D1**–**D3**) that were labelled with TMRM at resting conditions (incubation in AMES medium). (**A1**,**B1**,**C1**,**D1**) show the TMRM signals; (**A2**,**B2**,**C2**,**D2**) the phase contrast images. The signals from (**A1**–**A3**,**C1**–**C3**) and (**B1**–**B3**,**D1**–**D3**) were overlaid in (**A3**,**B3**,**C3**,**D3**). In (**C1**–**C3**,**D1**–**D3**), the respective slices were incubated with the mitochondrial uncoupling agent CCCP to verify the mitochondrial membrane potential as the specific source of the TMRM signals. (**E**) Quantification of the TMRM signals in the OPL of MOG/CFA-injected animals in comparison to CFA-injected control mice. MOG/CFA values were normalised to the arithmetic means of the CFA values, which were set to 100% in each independent experiment. Data are depicted as SuperPlots. The SuperPlots show the arithmetic means (green horizontal lines) ± S.E.M.s. Filled colored circles represent the arithmetic means of the individual independent experiments; the open circles show the individual datapoints from all experiments. Statistical analysis in (**E**) was performed with Wilcoxon signed-rank test. *p* < 0.05 was considered statistically significant. Abbreviations: OS, outer segment; IS, inner segment; OPL, outer plexiform layer; ONL, outer nuclear layer; N = number of independent experiments; n = number of analysed images; *, *p* < 0.05. Scale bars: 50 μm.

Therefore, we recorded mitochondrial membrane potential by using Tetramethylrhodamine and methyl ester (TMRM) in retinal slices from MOG/CFA-injected EAE mice and CFA-injected control mice on day 5 after injection (Figure 6). In conclusion, we observed a decreased TMRM signal in the OPL of MOG/CFA-injected mice under resting conditions in comparison to CFA-injected mice at day 5 after injection, indicating a decreased (i.e., depolarised) mitochondrial membrane potential in synaptic mitochondria of photoreceptor synapses in the OPL of MOG/CFA-injected EAE mice under resting conditions (Figure 6). Figure 6(A1–A3,B1–B3,C1–C3,D1–D3) show exemplary images; Figure 6E shows the quantification of the TMRM signals in the OPL, in which a decreased TMRM signal in the OPL of EAE mice is depicted.

**Figure 7:**
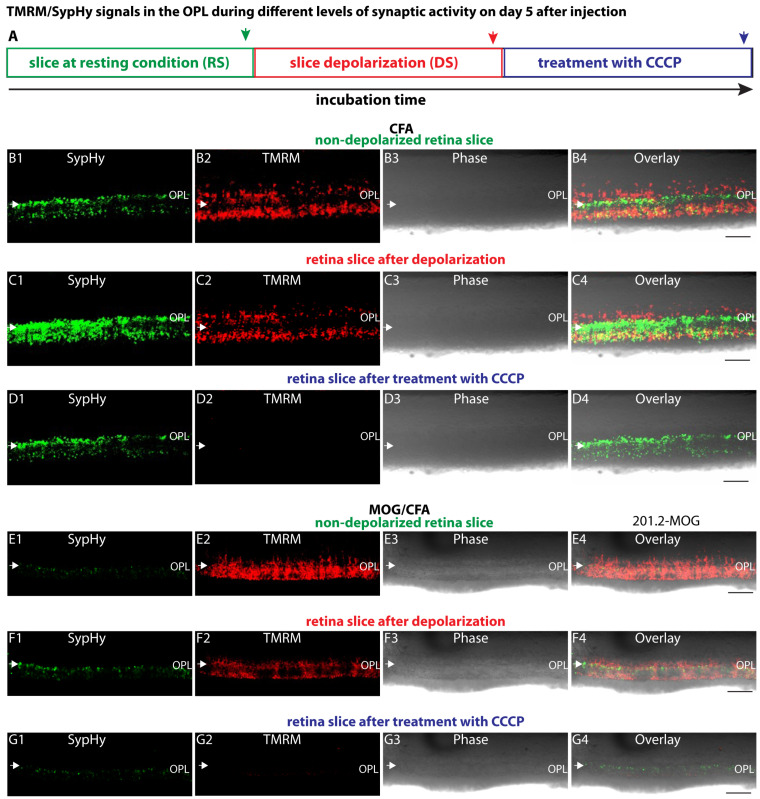

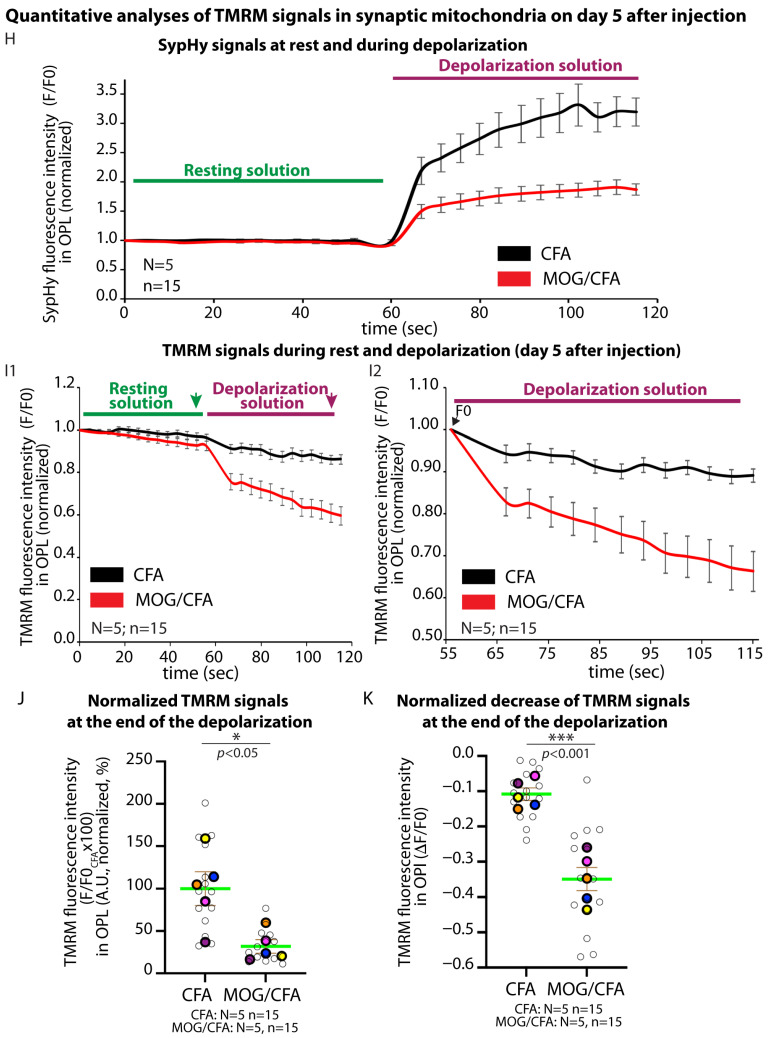
**(A–G) Representative images of TMRM fluorescence signals (mitochondrial membrane potential) and SypHy (exocytosis) signals in retinal slices from MOG/CFA-injected (EAE) SypHy mice and CFA-injected (control) SypHy mice obtained on day 5 after injection. Signals were recorded at different synaptic activity levels of the photoreceptor synapse in the OPL, i.e., during resting conditions, depolarisation and after treatment with CCCP.** (**A**) Schematic summary of the recording experiment. (**B1**–**B4**,**C1**–**C4**,**D1**–**D4**,**E1**–**E4**,**F1**–**F4**,**G1**–**G4**) Representative images of the corresponding living retinal slices from CFA-injected control mice (**B1**–**B3**,**C1**–**C3**,**D1**–**D3**) and MOG/CFA-injected EAE mice (**E1**–**E3**,**F1**–**F3**,**G1**–**G3**) at the indicated incubation conditions. (**B1**,**C1**,**D1**,**E1**,**F1**,**G1**) show the SypHy signals in the green channel; (**B2**,**C2**,**D2**,**E2**,**F2**,**G2**) show the TMRM signals in the red channel; the phase contrast images are shown in (**A3**,**B3**,**C3**,**D3**,**E3**,**F3**,**G3**). The signals from (**B1**–**B3**,**C1**–**C3**,**D1**–**D3**,**E1**–**E3**,**F1**–**F3**,**G1**–**G3**) were overlaid in (**B4**,**C4**,**D4**,**E4**,**F4**,**G4**). In (**D1**–**D4**,**G1**–**G4**), the respective slices were incubated with the mitochondrial uncoupling agent CCCP to verify the mitochondrial membrane potential as a specific source of the TMRM signals. The colored arrows in (**A**) indicate the time points at which the respective signals were used for quantification see (**H**–**K**). Abbreviations: OPL, outer plexiform layer; RS, resting solution; DS, depolarisation solution. Scale bars: 50 μm. (**H**–**K**) **Decreased TMRM fluorescence signals in the OPL of MOG/CFA-injected EAE mice in response to depolarisation on day 5 after injection, indicating enhanced dissipation of mitochondrial membrane potential in synaptic mitochondria of EAE mice.** (**H**) The line graphs show the SypHy signals of the indicated mice recorded from the OPL during incubation in resting solution (in the first 60 s of incubation) and during depolarisation induced by adding a high K^+^-containing depolarisation solution (DS) (in the following 60 s). (**I1**) Same incubations as (**H**), but showing the TMRM signals recorded during resting conditions and during depolarisation. In (**I2**), TMRM values during depolarisation were normalised to the starting point of the depolarisation (F0, set to 1.00). (**J**,**K**) Quantification of the TMRM signals in the OPL of MOG-/CFA-injected animals in comparison to CFA-injected control mice under depolarisation. Data in (**J**,**K**) are depicted as SuperPlots. The SuperPlots show the arithmetic means (green horizontal lines) ± S.E.M.s. Filled circles represent the arithmetic means of the individual independent experiments; the open circles show the individual datapoints of all experiments. (**J**) shows normalised TMRM signals at the end of depolarisation. MOG/CFA values are normalised to the arithmetic mean of all CFA experiments (the latter set to 100%). (**K**) displays the depolarisation-induced decrease in TMRM signals normalised to F0. Statistical analyses in (**J**,**K**) were performed with Welch’s *t*-test (*t*-test with Welch’s correction). *p* < 0.05 was considered statistically significant. Abbreviation: CFA, complete Freund’s adjuvant; CCCP, carbonyl cyanid-m-chlorphenyl hydrazon; DS, depolarisation solution; MOG, myelin oligodendrocyte glycoprotein; EAE, experimental autoimmune encephalomyelitis; OPL, outer plexiform layer; S.E.M., standard error of the mean; TMRM, Tetramethylrhodamine, methylester; N = number of independent experiments; n = number of analysed images; *, *p* < 0.05; ***; *p* < 0.001.

Next, we analysed the TMRM signals during synaptic activity. Synaptic activity, i.e., exocytosis of synaptic vesicles, was induced by depolarisation and monitored with transgenic SypHy mice, as previously described [75,78]. The enhanced SypHy responses in the OPL of the respective retinal slices after adding the depolarisation solution demonstrated that the depolarisation successfully resulted in enhanced exocytosis of synaptic vesicles at the photoreceptor synapses (Figure 7(B1,C1,E1,F1); see also Figure 7H for quantification). The depolarisation-induced enhancement of the SypHy signal in the OPL was stronger in CFA-injected control mice than in MOG/CFA-injected EAE mice (Figure 7(B1,C1,E1,F1); for quantification, see Figure 7H), similarly to what was previously described for experiments performed on day 9 after injection [75].

Depolarisation of the retinal slices resulted in a decrease in mitochondrial membrane potential of synaptic mitochondria in the OPL of MOG/CFA-injected EAE mice and CFA-injected control mice (Figure 7(B2,C2,E2,F2); for quantification, Figure 7(I1,I2,J,K)). Please note that the baseline TMRM resting values in the recordings in Figure 7(I1) were normalised to the starting point of the recording in both CFA-injected mice and MOG/CFA-injected mice. In Figure 7(I2), normalisation of the TMRM depolarisation signals in both CFA- and MOG/CFA-injected mice to the respective TMRM value at the end of the baseline recording (end of incubation in RS; set to 1.00 = F0; green arrow in Figure 7A) revealed that the drop in mitochondrial membrane potential in response to depolarisation was much stronger in synaptic mitochondria in the OPL of MOG/CFA-injected EAE mice in comparison to CFA-only-injected control mice (Figure 7J,K).

## 3. Discussion

Sufficient energy production is crucial for synaptic transmission, and mitochondria are considered to be essential for this process [30,46,47,102,103,104]. Failures in energy production and dysfunctions of mitochondria are suspected to be involved in MS [48,49,50,51,52,55,56,57,58,59,60,61,62,63,64,65]. Mitochondria might play an important role in the previously observed early synapse dysfunctions in the retina in the EAE mouse model of MS [84].

The precise role of mitochondria in the presynaptic terminals for synapse function and dysfunction is not fully understood [46]. In the CNS, not all presynaptic terminals contain mitochondria [105,106], possibly due to space restrictions, particularly in small presynaptic terminals, differences in mitochondrial axonal transport, or synapse-specific mechanisms. Rod photoreceptor synapses do contain a single large mitochondrion in their presynaptic terminal close to the presynaptic neurotransmitter release site. The presynaptic neurotransmitter release site of rod photoreceptor synapses is characterised by the presence of a particularly large synaptic ribbon that binds many synaptic vesicles to promote continuous neurotransmission at this synapse that requires efficient vesicle replenishment. Presynaptic mitochondria are also found in bipolar ribbon synapses in the inner retina close to the synaptic ribbon [107].

In a previous study, we demonstrated that retinal mitochondria were differentially affected in the early pre-clinical stages of EAE [84]. Synaptic mitochondria in photoreceptor presynaptic terminals were strongly altered in their protein composition (i.e., decreased MIC60 expression in synaptic mitochondria) at day 9 after injection, whereas mitochondria within the same cell, but in a different compartment (i.e., the inner segment), were not compromised in their MIC60 enrichment [84]. This is in line with previous studies that observed a functional diversity of mitochondria, depending on their subcellular localisation [108,109,110,111,112,113,114,115,116,117,118,119,120,121,122].

In the present study, we built upon these previous findings and analysed whether synaptic mitochondria were altered even at earlier time points in pre-clinical EAE and, if so, whether these very early changes are associated with functional changes in mitochondria and with visual performance. We found no changes in the MIC60 protein composition of photoreceptor mitochondria in the OPL at day 3 after injection. The absence of changes in MIC60 expression at synaptic mitochondria in the OPL correlated with a lack of changes in the optometry experiments in which we observed an unchanged contrast frequency at day 3 after injection. In contrast, the MIC60 enrichment in synaptic mitochondria was decreased already on day 5 after injection, concomitant with a decrease in visual performance. Remarkably, in addition, the immunosignal of RIBEYE, the main component of synaptic ribbons [123,124], is already decreased in photoreceptor synapses of the OPL at that early time point. A decrease in RIBEYE immunosignals (and a decrease in synaptic ribbon size) was already previously observed to occur on day 9 after injection [75,78]. This study demonstrated that synaptic changes in EAE already commence at an even earlier time point of pre-clinical EAE than previously observed. Since the mitochondrial pathology started on day 5 after injection, based on the described morphological and functional alterations, we further analysed the functional properties of photoreceptor synaptic mitochondria at this time point. For this purpose, we measured mitochondrial membrane potential with TMRM.

Already during resting conditions, we found a diminished mitochondrial membrane potential in MOG/CFA-injected EAE mice in comparison to CFA-injected control mice at day 5 after injection by quantitative analyses of the TMRM signals (Figure 6). In these experiments, the mitochondrial TMRM signals in the OPL were normalised to the total protein contents of the slices in these analyses to exclude an impact of slice thickness on the TMRM signals.

Similarly, depolarisation of retinal slices by a high K^+^-containing depolarisation solution led to a stronger drop (depolarisation) of mitochondrial membrane potential (MMP, ΔΨ_m_) in MOG/CFA-injected EAE mice than in CFA-injected control littermate mice (Figure 7). The decreased mitochondrial membrane potential during rest and during depolarisation is functionally relevant because the mitochondrial membrane potential is a crucial determinant for mitochondrial energy production [47,125,126,127]. Mitochondria generate ATP via oxidative phosphorylation, and the mitochondrial membrane potential is the key driving force in this process [47,125,126,127]. Thus, synaptic mitochondria display an altered mitochondrial membrane potential both under resting conditions and during increased synaptic activity in EAE mice at day 5 after injection (in comparison to the control mice).

As mentioned, the maintenance of the synaptic vesicle cycle is a highly energy-demanding process that depends on an appropriate supply of ATP, and this likely particularly applies to the tonically active ribbon synapses [26,30,33,34,35,128,129,130,131,132]. A decreased mitochondrial membrane potential in the single mitochondrion of rod photoreceptor ribbon synapses can be expected to lead to a decreased production of ATP and, in consequence, to a reduction in synaptic vesicle cycling and a decrease in synaptic strength. In agreement with this thinking, we previously observed decreased synaptic vesicle exocytosis on day 9 and 7 after injection [75,78], and, in this study, even earlier, i.e., at day 5 after injection. Interestingly, the Drosophila *fratboy/drp1* mutant is characterised by the absence of mitochondria from synapses [133]. This mutant shows a defect in recruiting synaptic vesicles from the reserve pool, particularly at high levels of stimulation, a defect that is partly rescued by the addition of ATP [133,134,135,136].

The activity of the synaptic vesicle cycle in most CNS synapses is dynamic and includes phases of higher and lower synaptic activity. Different activities in synaptic transmission will result in different demands on energy supply by mitochondria. Mitochondria are highly dynamic, versatile organelles and adjust their ATP production in response to increased synaptic activity and increased energy demands via an increase in mitochondrial Ca^2+^ concentration [30,36,133,136,137,138,139,140,141,142,143,144,145,146,147,148]. Ca^2+^ can enter the mitochondrial matrix via the mitochondrial Ca^2+^ uniporter (MCU) along its electrochemical gradient [122,146,149,150,151,152,153,154].

Short-term increases in Ca^2+^ in the presynaptic mitochondria, e.g., as a result of enhanced synaptic activity and depolarisation-induced presynaptic Ca^2+^ entry into the presynaptic terminal, will promote mitochondrial ATP production to support synaptic neurotransmission. The activity-induced enhanced mitochondrial ATP production will cover the energy requirements during increased synaptic activity [30,36,133,137,139,140,155,156,157]. This well-known physiological mechanism adjusts mitochondrial ATP production to different levels of synaptic activity and the respective energy demands and can be expected to be particularly important in the tonically active retinal ribbon synapses. The single large presynaptic mitochondrion in rod photoreceptor synapses close to the synaptic ribbon is ideally placed to respond to physiological fluctuations of synaptic activity that occur in response to depolarisation-induced opening of L-type voltage-gated Cav-channels that are placed at the base of the synaptic ribbon and other activity- and Ca^2+^-induced presynaptic events. Similarly, this synaptic ribbon-near presynaptic mitochondrion can likely also influence and shape presynaptic activity under physiological conditions, e.g., by its Ca^2+^-buffering properties.

In contrast, chronic Ca^2+^ overload of mitochondria leads to the opening of the mitochondrial permeability transition pore (mPTP), mitochondrial membrane potential depolarisation and further mitochondrial damage [122,153,155,158,159,160,161,162,163,164].

Previous studies demonstrated chronically elevated basal Ca^2+^ levels in photoreceptor ribbon synapses during resting conditions in EAE mice [76,165]. Presynaptic basal Ca^2+^ levels were measured on days 7, 8 and 9 after injection and showed similar levels of chronically elevated presynaptic cytosolic Ca^2+^ [76,165]. We speculate that these previously observed chronically elevated basal presynaptic cytosolic Ca^2+^ levels in photoreceptor synapses lead to mitochondrial damage in photoreceptor synapses, which was identified in the present study by a decrease in mitochondrial membrane potential and by a decreased enrichment of synaptic mitochondria with the functionally important protein MIC60 in photoreceptor synaptic mitochondria in the OPL in very early stages of pre-clinical EAE.

The decreased expression of MIC60 at synaptic mitochondria and the decreased mitochondrial membrane potential at synaptic mitochondria in EAE consistently demonstrate an early impairment of synaptic mitochondria of photoreceptor synapses. This correlation does not mean, though, that these mitochondrial phenotypes are directly causally linked to each other. Whether they are causally linked to each other remains to be elucidated. In other systems, MIC60 and mitochondrial membrane potential indeed appear to be directly linked to each other [166]. But this remains to be shown for the retinal synapses.

Similar to the retina, mitochondria in other systems were also found to be morphologically and functionally diverse [108,109,110,111,112,113,114,115,116,117,118,119,120,121,122]. Interestingly, synaptic mitochondria were observed to be more susceptible to Ca^2+^ overload than non-synaptic mitochondria [166,167], possibly reflecting the fact that synaptic mitochondria are directly exposed to synaptic activity-induced elevations of presynaptic Ca^2+^ and the resulting metabolic and homeostatic challenges. Additionally, they might also differ in mitochondrial Ca^2+^ transport mechanisms and mitochondrial proteins, such as cyclophilin D, an important regulator of the mitochondrial permeability transition pore, or SIRT3, a metabolic regulator of mitochondrial metabolism [167,168,169,170]. An enhanced susceptibility of synaptic mitochondria to Ca^2+^ overload can be particularly relevant for the continuously active photoreceptor ribbon synapses and explain their very early disease involvement in synaptic EAE in the retina.

## 4. Materials

### 4.1. Mice, Including Animal Care, Monitoring and Ethical Approval

As in our previous studies [75,76,78,81,84], we used the Experimental Autoimmune Encephalomyelitis (EAE) mouse model of multiple sclerosis. The EAE animal model is the most frequently used animal model of MS, and it reproduces many aspects of the neuroinflammatory events [12,13,14,15,16,17,18,19,20,21,22,23,24]. For EAE induction, healthy, non-pregnant 8–12 weeks old female C57BL/6J wildtype mice (Charles River) and transgenic SypHy reporter mice for exocytosis (on a C57BL/6J background, [75]) with a body weight between 20 and 25 gm were used, as indicated in the respective experiments. Only female mice were used in the present study for better comparability with our previous studies [75,76,78,81,84] and in order to appreciate sex differences in EAE. Mice were kept in the animal facility at the Institute of Experimental Surgery, Medical School, Saarland University, on a 12 h light–dark cycle and provided with standard food and water ad libitum. The laboratory mice lived in an enriched cage environment with mouse houses for shelter/hideaway and wood sticks (100% natural, untreated and pesticide-free) for playing. Animal health was regularly monitored in-house by local independent veterinarians of the animal facility to verify mouse health and to treat potential health problems. No adverse effect on overall mouse health was observed in the study. The Arrive guidelines 2.0 (https://arriveguidelines.org/resources/author-checklists, accessed on 5 January 2026) were appreciated. All animal procedures took place within the framework of the German/European animal protection law. All animal procedures and all animal experiments were reviewed, approved and monitored by the responsible animal authorities (Animal welfare officer (Tierschutzbeauftragte) of Saarland University and Landesamt für Verbraucherschutz; Geschäftsbereich 3; 66115 Saarbrücken, Germany). Animal experiments were performed under the animal experimentation permit GB3-2.4.2.2-25-2020 of the Landesamt für Verbraucherschutz, Geschäftsbereich 3, 66115 Saarbrücken, Germany.

### 4.2. Antibodies

All antibodies used in the present study have been previously characterised with the mouse retina [75,76,78,81,84]. Primary antibodies are summarised in Table 1; secondary antibodies are summarised in Table 2.

### 4.3. Solutions

-AMES medium [176,177,178].

AMES medium powder (with L-glutamine, without sodium bicarbonate) was purchased from Sigma-Aldrich (Taufkirchen, Germany, A1420). To make AMES medium, 8.8 g of the AMES medium powder (Sigma) and 1.9 g of sodium bicarbonate were dissolved in 900 mL of H_2_O. The pH was adjusted to pH 7.4 and finalised to a volume of 1 L with H_2_O. AMES medium was filter-sterilised and stored in 50 mL aliquots at 4 °C until use. Prior to use, all solutions for live recording were equilibrated to 37 °C (osmolality of 300–310 mOsmol/kg).

-Resting solution (RS) [75,76], containing the following: 132 mM NaCl, 3 mM KCl, 1 mM MgCl_2_ × 6H_2_O, 2 mM CaCl_2_, 10 mM HEPES, pH 7.4, 10 mM sodium pyruvate, and 10 mM D-glucose (osmolality 305–315 mOsmol/kg).

-Depolarisation solution (High K^+^ solution)

Depolarisation solution, as previously described [75,76], containing the following: 85 mM NaCl, 50 mM KCl, 1 mM MgCl_2_ × 6H_2_O, 2 mM CaCl_2_, 10 mM HEPES, pH 7.4, 10 mM sodium pyruvate, and 10 mM D-glucose (osmolality 305–315 mOsmol/kg). This solution is served as a 2× stock solution.

Study-specific chemicals:

#### 4.3.1. Tetramethylrhodamine and Methyl Ester (TMRM)

Tetramethylrhodamine and methyl ester (TMRM) was obtained as a lyophilised powder in 25 mg aliquots (Cat. No. I34361, T668, Thermo Fisher, Karlsruhe, Germany). An amount of 10 mM and 100 μM stock solutions of TMRM were prepared in DMSO. TMRM stock solutions in DMSO were stored at −20 °C. From the 100 μM TMRM stock solution, a final concentration of 50 nM TMRM in AMES medium was prepared as a working solution. The 50 nM TMRM working concentration was previously experimentally tested and determined as an optimal concentration for the recording of mitochondrial signals in retinal slices at the described conditions. Lower concentrations of TMRM produced only too weak signals, and higher concentrations of TMRM produced considerable background signals. The optimised TMRM final concentration applied in the present study is within the concentration range that was also used in other studies [179]. At this nanomolar concentration, TMRM can be expected to work in the non-quenching mode [107]. TMRM has an excitation maximum at ~543 nm and emits fluorescence light in a window between ~565 nm and ~605 nm. For TMRM excitation, the 561 nm laser line of the A1R confocal microscope was used. The TMRM signal was detected with an A1-DU4 4-channel Galvano detector unit equipped with a 595/50 band pass filter for spectral selection.

#### 4.3.2. Carbonyl Cyanid m-Chlorphenyl Hydrazon (CCCP)

A 50 mM CCCP (Thermo Fisher Cat. No.: L06932.ME) stock was made with DMSO as solvent. From this stock, an 80 μM working stock was diluted in the respective medium indicated in the experiments. This working stock was diluted 1:1 with the respective indicated medium in the blocking experiment to produce a final CCCP concentration of 40 μM.

## 5. Methods

### 5.1. EAE Induction in Mice

EAE induction was performed exactly as previously described [75,76,78,81,84]. For EAE induction, a total of 200 µg of MOG_35–55_ peptide (MEVGWYRSPFSRVVHLYRNGK) was injected subcutaneously into the axilla and groin of the experimental EAE mice. The peptide was thoroughly suspended in complete Freund’s adjuvant (CFA) before injection. Mice that were injected with the same volume of CFA only served as negative control mice. On the same day, ~1–2 h after MOG/CFA or CFA only injection, and on the next day, both EAE and control mice were injected intraperitoneally with pertussis toxin (PTX) (List Biological Laboratories via Biozol (Eching, Germany #181)). A total of 200 ng PTX was used for each injection, as previously described [75,76,78,81,84]. MOG/CFA-injected EAE mice and CFA-injected control mice were housed in the same cage under identical conditions to minimise environmental confounding. Mice were randomly (by chance) allocated to experimental or control groups and housed in the same cage to minimise environmental confounding. The person who randomly allocated the mice to the experimental or control group was different from the person who analysed the mice. The person analysing was blinded to the identity of the mice. Animals were analysed at the indicated time points/days after MOG/CFA injections for EAE mice (or CFA only injections for control mice) in the pre-clinical phase of EAE on day 3 and day 5 after injection (as indicated in the respective figures). In the pre-clinical phase of EAE, no scorable symptoms (no paresis/no paralysis) as mentioned in the standard scoring sheets of the Institutional Animal Care and Use Committee (IACUC) of Wayne State University (https://research.wayne.edu/iacuc/eaerodentssop, accessed on 5 January 2026) were present. These early time points (i.e., day 3 after injection and day 5 after injection) were chosen to minimise the possibility that the synaptic changes could be secondary consequences of alterations in the white matter/optic nerve, e.g., of demyelination.

### 5.2. Embedding of Retinal Samples in Epoxy Resin for Immunocytochemistry

Embedding of retinal samples into epoxy resin was done exactly as previously described [75,76,78,81,84,180]. From the polymerised epoxy blocks, 0.5 μm thin sections were cut with a Histo diamond knife (Diatom, Nidau, Switzerland) and an UltraCut E ultramicrotome (Reichert-Jung, Nußloch, Germany).

### 5.3. Processing of Semi-Thin Retinal Resin Section for Immunocytochemistry

Processing of semi-thin resin sections was performed, as previously described [75,76,78,81,84,180]. After removing the epoxy resin with 30% Na-Methylate (in methanol) solution (Sigma/Merck, Taufkirchen, Germany, #8.18194.0100), xylene/methanol 1:1 (*v*/*v*), acetone (2×), H_2_O and phosphate-buffered saline (PBS) (10–13 min each step), sections were incubated with the primary antibody dilutions, as mentioned in Table 1. For double-immunolabelling experiments, the two primary antibodies were applied simultaneously (overnight (ON), 4 °C). The two different primary antibodies were raised in two different animal species, as indicated in the respective experiments. The next day, the unbound primary antibody was removed by several washes with PBS. Binding of the primary antibodies was detected with the corresponding secondary antibodies and antibody dilutions, given in Table 2. Incubation in the fluorophore-conjugated secondary antibodies was performed at room temperature (RT) for 1 h. Secondary antibody was removed by several washing steps with PBS. Immunolabelled sections were mounted in N-propyl gallate (NPG) antifade, as previously described [75,76,78,81,84,180]. As a negative control, the primary antibody was omitted, with all other steps being the same. The negative control served to detect autofluorescence or non-specific, primary antibody-independent fluorescence signals. In each experiment, at least 3 independent pairs of MOG/CFA-injected EAE mice and CFA-injected control mice, but mostly 5 independent pairs, were used for immunolabelling, as indicated in the specific experiments.

### 5.4. Confocal Microscopy

Confocal microscopy was performed with an A1R confocal microscope (A1R, Düsseldorf, Germany), as previously described [75,78,81,84,179,180,181,182]. Images of fixed samples/immunolabelled resin sections were acquired with a 60× Plan-Apochromat oil immersion objective (NA 1.4) (Nikon, Düsseldorf, Germany). For recordings of mitochondrial membrane potentials in living mouse retina slices, a 10× water dipping objective (NA 1.2, 0.3WD, DIC N1) was used with laser excitation wavelengths of 488 nm and 561 nm. Image acquisition was controlled with the NIS Elements software (Nikon, Düsseldorf, Germany).

### 5.5. Quantification of Immunosignals of Photoreceptor Synapses in the Outer Plexiform Layer

For quantification, confocal images obtained from the A1R confocal microscope were exported as TIFF files (8-bit). The region of interest (ROI) was set by drawing a rectangular area of interest along the entire outer plexiform layer (OPL) with the ROI manager of NIH ImageJ software (Fiji, version 1.48F; [183,184]). The rectangular ROI, which covered the entire OPL, was determined with the help of the characteristic actin or RIBEYE immunosignals, as previously described and as specified in the respective experiments [75,76,78,81,84]. The split channel option was used to apply the same rectangular ROI to the proteins of interest in the OPL, and the integrated densities of the respective immunosignals were measured. Identical ROIs were used for CFA and MOG/CFA samples by selecting the same ROI in the Analyse–tools–ROI manager in ImageJ (Fiji version 1.48F; [183,184]). Using Analyse–tools–ROI manager in NIH ImageJ (Fiji, version 1.48F), the same identical rectangular ROI was chosen for all images in the same experiment (MOG/CFA- vs. CFA-injected mice). The split-channel option was used to apply the same rectangular ROI to the different proteins of interest in the double-immunolabelling experiments [185,186]. Each experiment has been repeated at least 3 times, but mostly 5 times, in a blinded manner with the experimenter not knowing whether the sample was from a CFA- or MOG/CFA-injected animal. To analyse the immunofluorescence signals, the integrated density in the ROI of all images was analysed. The arithmetic mean was determined for each experimental group (CFA or MOG/CFA) in Microsoft Excel. For better comparison of different pairs, the IF signal (integrated density) values of MOG/CFA were normalised to the CFA arithmetic mean value, which was normalised/set to 100% [75,76,78,81]. Statistical significance testing was done on GraphPad 10.6.1 Prism, as described below.

### 5.6. Measuring Mitochondrial Membrane Potential with Tetramethylrhodamine and Methylester (TMRM)

#### 5.6.1. Measuring Mitochondrial Membrane Potential with TMRM: General Principle

Tetramethylrhodamine and methyl ester (TMRM; Thermo Fisher, Karlsruhe, Germany; T668) is a lipophilic membrane-permeable cationic potentiometric fluorescent dye, which allows for the measurement of the mitochondrial membrane potential (ΔΨ_m_) in living mitochondria [179,187,188,189]. Upon its penetration through the inner mitochondrial membrane, the positively charged TMRM accumulates inside the matrix of healthy active mitochondria driven by the excess negative charge on the matrix side of the inner mitochondrial membrane. The more negative the inner mitochondrial membrane potential is, the more TMRM is trapped, and the stronger the TMRM signal of the respective mitochondria is if the TMRM is loaded at lower nanomolar concentrations. Consequently, the mitochondrial TMRM signal will be stronger/brighter if the inner mitochondrial membrane has established a steep proton gradient across the inner mitochondrial membrane, thus reaching a highly negative, hyperpolarised mitochondrial membrane potential ΔΨ_m_. In contrast, mitochondrial depolarisation (i.e., ΔΨ_m_ becoming less negative, collapse of mitochondrial membrane potential) will result in decreased TMRM signals. Carbonyl cyanide-4-phenyl hydrazone (CCCP) depolarises the inner mitochondrial membrane potential by acting as a protonophore that dissipates the proton gradient across the inner mitochondrial membrane [190].

#### 5.6.2. Preparation of Slices from the Dissected Mouse Retina for TMRM Recording

The recording of mitochondrial membrane potential with TMRM was performed with the same animals that had been previously analysed in the optometry tests (see below), to judge the visual performance of these mice also on a systems level for visually guided behaviour. For recording of mitochondrial membrane potential from retinas of MOG/CFA-injected EAE mice and CFA-injected control mice, living retinal slices were prepared as previously described [75,76,78,84]. In these experiments, mouse retinas were processed in a blinded manner with the experimenter not knowing whether the respective retina was from a MOG/CFA-injected EAE mouse or from a CFA-only-injected control mouse.

Dissected retinas were first transferred into a drop of AMES medium, which supports the health of the retinal tissue, and gently flattened within the AMES medium (A1420, Sigma-Aldrich, Taufkirchen, Germany) by four incisions into the retina, as described before [75,76,78,84]. Next, retinas were transferred to a gridded nitrocellulose membrane (13 mm diameter, 0.45 µm^2^ pore size; Millipore, Darmstadt, Germany; #HABG01300), which was positioned on a silica sieve funnel. The retina on the nitrocellulose membrane was further flattened by applying suction to the nitrocellulose membrane via a connected 20 mL syringe (20× manual suction by pulling the syringe plunger) to promote better adherence of the attached retina to the filter membrane. After suction, the filter membrane containing the retina was fixed on top of a glass coverslip with Vaseline streaks at the border, with the retina facing up. The retina was cut into ~200–~300 µm thick slices (~10–12 slices) by a Werblin-tissue slicer, as previously described [75,78]. Every single slice was fixed perpendicularly between two thin parallel streaks of Vaseline on the top of a round coverslip (25 mm diameter), and the space in between the two parallel streaks was filled with 200–300 μL of AMES medium. In this way, the retinal ganglion cells are positioned close to the filter membrane, and the photoreceptor cell layer is positioned at the “free” surface of the assembly. Next, all slices were incubated throughout the experiment at 29 °C in a 5% CO_2_ cell culture incubator.

Recording of mitochondrial membrane potential with TMRM was done on day 5 after injections. To record baseline mitochondrial membrane potential (mitochondrial membrane potential “at rest”), we used retinas from C57BL/6J littermate mice that were either injected with MOG/CFA (EAE mice) or with CFA only (control mice). For recording mitochondrial membrane potential during depolarisation, we used transgenic SypHy reporter mice for exocytosis [75]. SypHy mice were used to get an additional readout to verify the success of depolarisation on exocytosis, as previously described [75].

#### 5.6.3. Recording of Mitochondrial Membrane Potential in Photoreceptor Synapses at Resting Conditions with TMRM

For TMRM recording, the retinal slice was washed three times with AMES medium (pH 7.4) and incubated with 300 μL of 50 nM TMRM dye diluted in AMES medium (100 μM in dimethyl sulfoxide (DMSO) is an original stock solution for stability) for 15 min in a cell culture incubator (29 °C, 5% CO_2_). At the end of the incubation period, the slice was washed three times with AMES medium to remove unbound TMRM dye. For recording, the coverslip containing the incubated slice was placed in the open system recording chamber and filled with 2.0 mL fresh carbogenated AMES media. The slice was adjusted to define the retinal layers, in particular the OPL, by using the phase contrast image obtained with the confocal microscope using a 10× water objective (N.A. 1.2, 0.3WD, DIC N1). Then, the TMRM fluorescence signal within the OPL, the region of interest (ROI), was recorded with the 561 nm laser line. For obtaining the TMRM baseline fluorescence signals, images from the retinal slices (obtained from CFA- or MOG/CFA-injected mice) were first captured at time point 0 (~2 s after the beginning of the recording with an exposure time of 100 ms). To validate the mitochondrial membrane potential as the specific source of TMRM signals and to discriminate mitochondria-dependent TMRM signals from potential background signals (e.g., tissue autofluorescence from non-mitochondrial sources), we performed blocking experiments with the mitochondrial uncoupling reagent CCCP. CCCP specifically dissipates mitochondrial membrane potential. For this purpose, 2.0 mL of the 80 µM CCCP stock solution, diluted in AMES medium, was added (without suction; after TMRM baseline recording) to the same volume of AMES medium in which the slices were incubated (to generate a final concentration of 40 µM of CCCP). Then, the recording was continued in the AMES/CCCP medium for 20 min at a temperature of 29 °C using a temperature regulator (Harvard Instruments).

The exposure time for each captured image was ~100 ms, and the images were captured every 4 s through the 20 min incubation time to continuously record the TMRM signals. At the end of the incubation in AMES/CCCP, the TMRM fluorescence intensity value obtained at ~19 min incubation time was used for background subtraction. During the recording, all camera settings were identical by using the “re-use camera setting” of the NIS Elements software (AR 3.2 64bit) of the confocal microscope. At the very end of the 20 min recording in AMES/CCCP, the recorded slice was dissolved in 60 µL SDS/Laemmli buffer for protein quantification and subsequent normalisation of the TMRM signal intensity to the total protein amount to decrease variability due to differences in slide thickness. Importantly, TMRM recording data of retinal slices were obtained from littermate pairs (CFA and MOG/CFA injected mice) in a blinded manner and in alternating fashion within 8–10 h to further minimise potential variability factors.

#### 5.6.4. Quantification of TMRM Fluorescence Baseline Signals in the OPL at Rest

The images were exported (in TIFF format, 8-bit) during baseline recording and after CCCP blocking. As described above, phase contrast images were always used to define the OPL as the region of interest (ROI). Using NIH ImageJ software (Fiji, version 1.48F), a freehand tool option was used to draw and define the OPL as an ROI in the phase contrast image. After using the split channel option of NIH ImageJ software (Fiji, version 1.48F), the correct placement of the ROI to the OPL was confirmed by the TMRM fluorescence signal in the red channel (obtained with the 561 nm laser excitation line). The integrated density of the TMRM fluorescence signal in the red channel and the defined area were measured at baseline (image captured at ~2 s after the beginning of the recording) and at the end of TMRM signal recording in AMES/CCCP (at ~19 min incubation in AMES/CCCP). Next, the integrated density of each slice, determined as described above with background subtraction for both time points (baseline and after CCCP blocking), was normalised to the corresponding area being measured. Then, the resulting value was normalised to the total protein amount of the slice (in µg), as quantified by the Amido Black method [191]. This latter normalisation procedure was done to determine the TMRM baseline signal (at the beginning of the recording) and the TMRM signal in the presence of the uncoupling agent CCCP (at the end of the incubation in AMES/CCCP at ~19 min of recording). Delta values were defined by subtracting the normalised blocking values from the normalised baseline values of the TMRM signal at rest (baseline recording, no depolarisation). The quantification was done identically for both MOG/CFA and CFA samples in a blinded manner, with the experimenter not knowing the identity of the samples. In these experiments, we used seven littermate mice from MOG/CFA (EAE experimental mice) and CFA-only immunised mice (control mice) at day 5 after injection. The normalised integrated density values from the OPL of MOG/CFA-injected mice were related to the normalised arithmetic mean of integrated density values from the OPL of CFA-injected mice (100%) using Microsoft Excel. Statistical analyses were done with GraphPad Prism version 10.6.1, as described below.

#### 5.6.5. Recording and Quantification of TMRM Signals in Photoreceptor Synapses of the OPL During Depolarisation in Retinas from SypHy Transgenic Reporter Mice on Day 5 After Immunisation

In order to verify the success of depolarisation on exocytosis of synaptic vesicles in photoreceptor synapses in the OPL, we used retinal slices from transgenic SypHy mice for recording, as previously described [75,78]. MOG/CFA-injected SypHy EAE and littermate CFA-injected control mice were analysed on day 5 after injection. Injections with MOG/CFA (EAE mice) or CFA only (control mice) were done exactly as described above.

Retinal slices from SypHy transgenic reporter mice were made as described above for C57BL/6J mice and as described in previous studies [75,76,78,84]. After slicing of the retinas, sections were incubated in 200–300 µL AMES medium in a 29 °C 5% CO_2_ incubator. To decrease the variability through the recording, one slice from either a MOG/CFA-injected experimental mouse or a CFA-injected control mouse was chosen in an alternate fashion. The identity of the slices, i.e., whether they were obtained from a MOG/CFA-injected mouse or a CFA-only-injected mouse, was not known to the experimenter. The slice selected for recording was incubated with 300 µL of 50 nM TMRM dye (final concentration) dissolved in AMES medium (15 min in a 5% CO_2_ incubator at 29 °C). After incubation with the TMRM solution, the slice was washed three times with resting solution (RS) in the circular-open recording chamber to remove excess TMRM dye. Afterwards, slices were submerged in 2.0 mL fresh carbogenated RS in the recording chamber. The recording chamber temperature was maintained at 29 °C by a temperature controller (Harvard Instruments) throughout the entire recording. Image acquisition was performed with a 10× objective water lens (N.A. 1.2, 0.3 W DIC N1).

The responses of the OPL at excitation were continuously recorded with the 488 nm laser excitation line (for measuring SypHy responses) and 561 nm laser excitation line (for measuring TMRM responses). All the recording settings were the same as in the baseline recording experiments, and the acquisition rate was 1 image every 4 s. An image was acquired in both channels with an exposure time of 100 ms for both channels. Recording conditions were always kept the same in experimental and control slices. Also, the camera settings were kept the same by using the “re-use camera setting” in the NIS acquisition software of the confocal microscope that controls the image acquisition. After the end of the slice recording, the slice was dissolved in 60 µL SDS Laemmli buffer for protein quantification with the Amido Black method [191] to correct for potential differences in slice thickness that could lead to differences in TMRM signals. The total protein amount of the recorded slices (in µg) was calculated. Five different independent TMRM optical recordings were performed under these conditions (recording of resting conditions followed by depolarisation) using five independent immunisations of MOG/CFA and CFA littermate mice.

TMRM fluorescence signals were continuously recorded from the OPL in three subsequent phases, i.e., first, during resting conditions to determine the baseline TMRM signals; second, subsequently, during depolarisation; and lastly, third, after treatment with the mitochondrial uncoupling agent CCCP that dissipates the mitochondrial membrane potential. First, the baseline SypHy/TMRM signal was measured during the first 60 s of recording when the slices were incubated with 2.0 mL resting solution (RS). Following the recording of the SypHy/TMRM baseline signals at resting conditions in RS, slices were depolarised for 60 s with depolarisation solution (DS), containing 25 mM KCl (final concentration). The depolarisation solution was obtained by manually adding an identical volume of 2× depolarisation solution (2xDS, containing 50 mM KCl) to the 2 mL of RS that already covered the slices. After depolarisation, DS was replaced first by 2 mL of RS to remove the depolarisation stimulus (60 s) and next by 2 mL of CCCP blocking solution (80 µM of CCCP in RS; final dilution 40 µM). Incubation in CCCP (in RS) was performed for 20 min. All solutions were added manually during continuous suction to keep the incubation medium at a total volume of ~2 mL.

#### 5.6.6. Quantification of TMRM Signals in the OPL During Depolarisation in Retinas from SypHy Transgenic Reporter Mice

The recording images obtained from the resting and depolarisation phase, as well as from the treatment with CCCP, were exported as TIFF files and imported into NIH ImageJ. Next, the region of interest (ROI), the OPL, was carefully assigned by using the contrast phase images and the green SypHy fluorescence signal (obtained with the 488 nm laser excitation line). The ROIs in the captured images were made with the freehand tool option of the NIH ImageJ software (Fiji, version 1.48F). Then, the identical ROI was used to measure the integrated density of the TMRM signal in the red channel (561 nm laser) in the recorded slice. In addition to the integrated density of the TMRM fluorescence signals, the area of the ROI was also measured. The same measurements and quantifications were done in the same slice during the three phases: (1) resting conditions in RS, (2) at the end of high K^+^-induced depolarisation and (3) at the end of the CCCP treatment. Finally, all data were exported to Microsoft Excel.

In each experiment, the analysis was done similarly for each slice. The measured integrated density was normalised to its corresponding area at each time point. Then, the resulting value was also normalised to the total protein amount (in µg). These analyses were done on all the images at all phases (baseline at resting conditions, at the end of high K^+^-induced depolarisation and after CCCP treatment).

For a better comparison of the effect of depolarisation between MOG/CFA and CFA injected mice, we did the following quantitative analyses: The arithmetic mean of the last three values during the baseline recording (in RS) was set to F_0_ (reference time point), and the arithmetic mean of the last three values at the depolarisation phase was set to F_dep_. From these values, the background that remained after CCCP treatment was subtracted. Please note that this step, i.e., the subtraction of the background remaining after CCCP treatment, was done for all values obtained during baseline recording in RS and during depolarisation. Then, the changes (Δ) of TMRM fluorescence signals before and after depolarisation were calculated for each slice using the equation (F_dep_ − F_0_)/F_0_ with Microsoft Excel. Statistical analysis was done using GraphPad Prism 10.6.1, as discussed below.

## 6. Acquisition of Visual Performance in Mice

Visual behaviour was assessed using a virtual optomotor system setup (OptoMotry; CerebralMechanics, Lethbridge, AB, Canada) [75,101] at day 3 or day 5 after induction of EAE. To determine visual acuity, first, the highest spatial frequency, which was able to provoke optomotor responses, was identified. Next, two different frequencies (both below the spatial threshold) were chosen to analyse the contrast sensitivity of the respective mice. For this, a slow speed grating (0.192 cycles/degree) with changing contrast and a medium speed grating (0.272 cycles/degree) with changing contrast were used to determine the contrast threshold.

Contrast sensitivity was assessed using the following equation:

$$\mathrm{contrast}\;\mathrm{sensitivity}\;=\frac{100\%}{\frac{\left(Lmax-Lmin\right)}{\left(Lmax+Lmin\right)}}\times \frac{1}{contrast\;threshold}$$

with *L_max_* (246.15 cd/m^2^) and *L_min_* (0.237 cd/m^2^) being the maximum and minimum illuminations of the computer screens.

Mice were tested during their daylight cycle (usually between 9:00 and 11:00 a.m.). The mouse to be analysed was placed on the platform of the recording chamber. Movement of the mouse head in response to the rotating grating provided by the optomotor software was tracked by the operator using a crosshair cursor superimposed on the live video image. If reflexive head movements of the mouse followed cylinder rotation, the examiner judged that the mouse could see the grating (positive response). If the mouse did not track the grating (negative response), it was judged that the grating could not be perceived by the mouse. Both types of responses were feed-backed to the system by the examiner in real time. In the case of frequency testing, the software switched randomly to the next frequency. In case of contrast threshold testing, spatial frequency was kept constant, and the software randomly changed the contrast of the grating in response to the examiner’s input. If measured repeatedly, mice were returned to their housing cages after each measurement with access to water and food ad libitum. Mice had at least 30 min breaks in between measurements. The examiner was blinded to the treatment of the mice.

## 7. Statistical Analyses

Statistical analyses were performed with GraphPad Prism 10.6.1. Each set of independent experiments contained both CFA-injected control animals as well as MOG/CFA-injected EAE animals analysed at the time points indicated in the respective experiments. Data presentation as SuperPlots and statistical analyses were done according to [192] with GraphPad Prism 10.6.1. In all quantification figures, the arithmetic means of the individual independent experiments, the global mean of all experiments (±S.E.M) and all individual datapoints were plotted accordingly, as specified in the respective figures. The specific statistical tests applied to the respective indicated datasets are given in the figure legends. Shapiro–Wilk tests were used to determine whether data were normally or non-normally distributed. Based on normal or non-normal distribution of the datasets, the indicated statistical tests for normally or non-normally distributed data were applied as specified in the corresponding figure legends. A *p*-value < 0.05 was considered statistically significant.

## 8. Conclusions

Our study supports a role of synaptic mitochondria malfunctions for retinal synapse pathology at very early steps by using the EAE mouse model of multiple sclerosis. EAE is the commonly used model system of multiple sclerosis and reproduces many symptoms also found in the human disease. It will be useful to address mitochondrial dysfunctions also in human multiple sclerosis patients at very early stages of the disease.

## Figures and Tables

**Table 1:** Primary antibodies.

Antibody	Source	References	Dilution
RIBEYE(B) domain (mouse monoclonal, clone 2D9)	Lab-made	[75,171]	1:1000 (IF)
MIC60/Mitofilin (affinity-purified rabbit polyclonal)	Proteintech, 10179-1-AP	[84,172,173,174]	1:1000 (IF)
Actin (mouse monoclonal antibody, clone C4), #1501R	Millipore #1501R	[175]	1:1000 (IF)

IF, immunofluorescence microscopy.

**Table 2:** Secondary antibodies.

Antibody	Source	Dilution
Chicken anti-mouse Alexa488	Invitrogen Molecular Probes, A-21200 (Thermo Fisher, Karlsruhe, Germany)	1:1000 (IF)
Donkey anti-rabbit Alexa568	Invitrogen, Molecular Probes, A-10042 (Thermo Fisher, Karlsruhe, Germany)	1:1000 (IF)
Chicken anti-rabbit Alexa488	Invitrogen, Molecular Probes, A-21441 (Thermo Fisher, Karlsruhe, Germany)	1:1000 (IF)
Donkey anti-mouse Alexa568	Invitrogen, Molecular Probes, A-10037 (Thermo Fisher, Karlsruhe, Germany)	1:1000 (IF)

IF, immunofluorescence microscopy.

## Data Availability

The original contributions presented in the study are included in the article, and further inquiries can be directed to the authors.
